# Congenital Disseminated Malignant Rhabdoid Tumor Mimicking a Vascular Lesion

**DOI:** 10.7759/cureus.58337

**Published:** 2024-04-15

**Authors:** Scott K Wang, Prabhath Mannam, Katerina Dukleska, Fabiola Balarezo

**Affiliations:** 1 Pathology and Laboratory Medicine, Hartford Hospital, Hartford, USA; 2 Medicine, University of Connecticut, Hartford, USA; 3 Pediatric Surgery, Connecticut Children's Medical Center, Hartford, USA

**Keywords:** ini1, therapy, smarcb1, vascular lesion, congenital disseminated malignant rhabdoid tumor

## Abstract

A congenital disseminated malignant rhabdoid tumor (MRT) is an exceedingly rare and aggressive pediatric cancer marked by the presence of malignant rhabdoid cells in various organs, including the brain, kidneys, and soft tissues, at birth. It is often detected prenatally or shortly post-birth. The malignancy's aggressiveness results in a bleak prognosis, offering limited treatment options and low survival rates. Early diagnosis and comprehensive medical intervention are crucial, but managing this condition is complicated by its rarity. We herein presented a case of a 37 and 1/7 week gestation male infant with a rapidly growing arm soft tissue mass within two weeks, diagnosed as an MRT. Post-delivery examinations revealed multiple lesions in the lungs, kidney, liver, and adrenal glands. Notably, chemotherapy yielded a significant improvement in the arm lesion, contrasting with other lesions showing a limited response. This observation suggests potential tumor heterogeneity, emphasizing the necessity of diverse therapeutic regimens. Our case underscores the complexities of congenital disseminated MRT, prompting a reevaluation of treatment strategies for enhanced efficacy in managing this challenging pediatric cancer.

## Introduction

Congenital solid tumors, a subset of non-hematologic cancers, are relatively uncommon in newborns. Malignant rhabdoid tumor (MRT) is an especially rare and aggressive malignancy, primarily impacting children under three to four years of age [1-2]. While it typically arises in the central nervous system (~65%) and kidney (~35%), cases in various soft-tissue sites have been documented [3-5]. Due to its scarcity and aggressiveness, there is no established standard of care, and the overall survival rate remains below 50% [6-7]. Characterized by unique morphology and *SMARCB1* (or rarely *SMARCA4*) gene inactivation [8], congenital disseminated MRT poses a specific clinicopathologic challenge. In this case report, we present a unique instance of congenital MRT in the right forearm, mimicking a congenital vascular lesion.

The partial article information was previously presented as a meeting abstract at the 2023 Society for Pediatric Pathology Fall meeting on October 6, 2023.

## Case presentation

**Figure 1:**
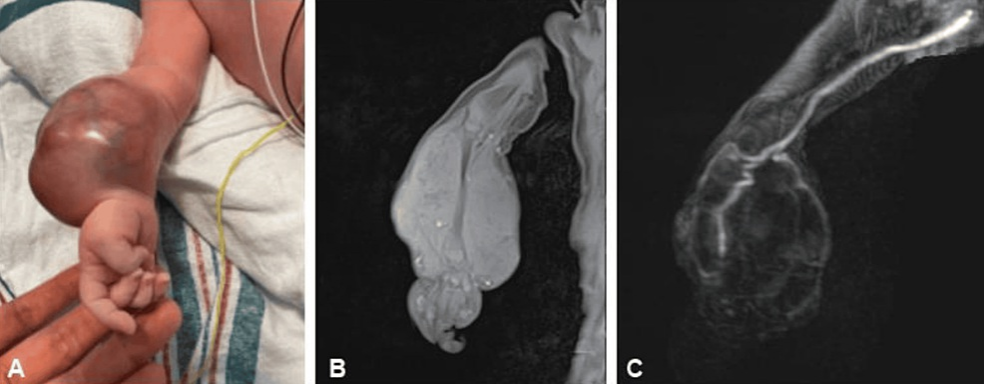
Soft tissue mass on the right forearm (A) that underwent T1-weighted MRI (B) and MRA (C) MRI: magnetic resonance imaging; MRA: magnetic resonance angiogram

During the prenatal ultrasound at 37 and 1/7 weeks gestation, a male infant was found to have a soft tissue mass in the right forearm that was not present in prior examinations. Given the rapid evolution of the finding, the baby was delivered via a cesarean section at 37 and 2/7 weeks. Initial physical examination revealed a vascular, bulbous, and taut soft tissue mass on the right forearm with a circumference of 19 cm (Figure 1A). An MRI of the right arm identified a 5.7 x 6.0 x 8.7 cm heterogenous mass that enveloped the radius and ulna (Figure 1B). A magnetic resonance angiogram (MRA) of the right arm then found the soft tissue mass to demonstrate hypervascularity with multiple phleboliths, increased flow within the mildly enlarged interosseus artery, and lateral displacement of the radial and ulnar arteries (Figure 1C). Given the findings from the initial physical examination and imaging of the right arm, the mass was suspected to be vascular in origin, with a leading differential diagnosis of Kaposiform hemangioendothelioma and epithelioid angiosarcoma.

**Figure 2:**
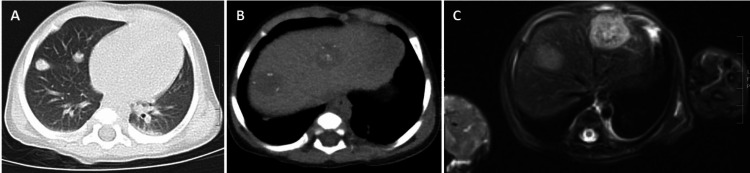
Multiple lesions are identified in the lung CT (A), liver CT (B), and MRI (C) CT: computed tomography; MRI: magnetic resonance imaging

An upper abdominal CT identified a 1.6 cm mass in the anterior aspect of the right lower lobe of the lung (Figure 2A). Additionally, three T2 bright hepatic lesions were found in the liver (Figure 2B-2C): a 2.4 cm mass in segment 2 of the left hepatic lobe, a 2.0 cm mass in segment 8 of the right hepatic lobe, and a 0.8 cm mass in segment 4A. At the time of the abdominal MRI, the patient was also found to have elevated aspartate aminotransferase, alanine aminotransferase, lactate dehydrogenase, and gamma-glutamyltransferase, all of which are consistent with the liver lesions identified on the abdominal MRI. The discovery of these lung and liver lesions modified the differential diagnosis to include metastatic disease as a possible etiology of the right arm soft tissue mass.

**Figure 3:**
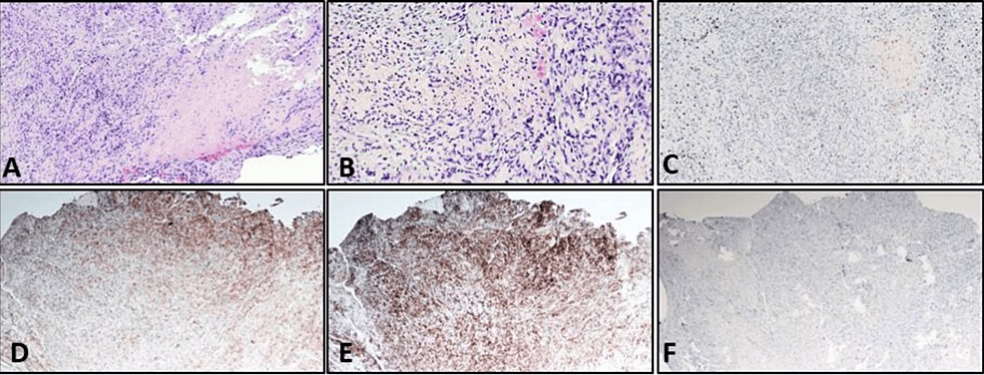
Lesion displays necrosis (A) A high power field shows polygonal cells with large eccentric nuclei, prominent nucleoli, pleomorphism, and mitosis (B). Tumor cells lose INI-1 nuclear expression (C) and are positive for EMA (D), CD56 (E), and focal SALL4 (F).

Five days after birth, a biopsy of the right arm mass was performed and sent for histopathological analysis and genetic sequencing. Histologically, the arm lesion exhibited polygonal cells with eosinophilic cytoplasm, large eccentric nuclei with prominent nucleoli, necrosis, moderate pleomorphism, and a high mitotic rate. Immunohistochemical analysis highlighted the loss of SMARCB1 nuclear expression. The biopsied lesion further demonstrated positive expression for EMA and CD56, with a few cells positive for CK AE1/3 and SALL-4. Expression of desmin, myogenin, CD34, S100, chromogranin, synaptophysin, and TFE3 was not detected, while SMARCA4 and H3K26 expression were retained (Figure 3). Next-generation sequencing identified a homozygous gene deletion of *SMARCB1*. Taken together, the histopathology, immunohistochemistry, and molecular studies established a diagnosis of extrarenal MRT.

**Figure 4:**
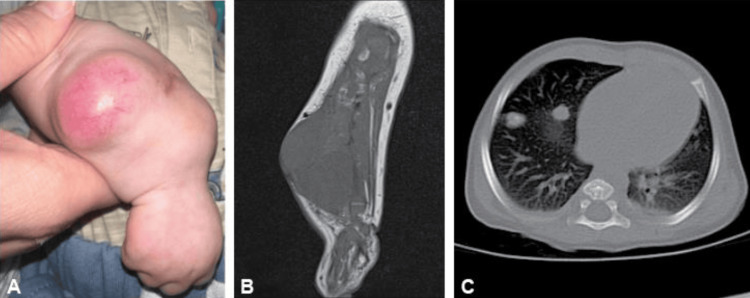
Soft tissue mass on the right forearm (A) that underwent T1-weighted MRI (B). A CT scan of the lungs identified multiple nodules (C) MRI: magnetic resonance imaging; CT: computed tomography

Prior to treatment initiation, the patient underwent an abdominal ultrasound, which revealed new hypoechoic lesions within both kidneys: a 0.7 cm lesion in the lower pole of the right kidney and a 0.7 cm lesion in the interpolar region of the left kidney. In addition, a 0.8 cm hypoechoic lesion was identified in the right adrenal gland. At the start of chemotherapy, the patient’s liver lesions had increased in size, and the arm lesion had grown to 23 cm in circumference. The patient was started on a chemotherapy regimen consisting of vincristine, doxorubicin, cyclophosphamide, carboplatin, and etoposide. Following 15 weeks of chemotherapy, the patient underwent repeat imaging to assess tumor responsiveness to the treatment. The patient’s right arm lesion decreased significantly in size (Figure 4A), and on MRI, it was measured at 4.2 x 5.2 x 6.5 cm. However, it demonstrated multiple areas of cortical destruction and infiltration of the middle and distal radius (Figure 4B). An abdominal MRI revealed the continued presence of hepatic, adrenal, and renal lesions, with non-significant changes in size. A CT of the chest identified a total of sixteen bilateral pulmonary nodules in various lobes, indicative of progressive metastatic disease (Figure 4C). The patient’s family opted to discontinue treatment, and the patient passed away at the age of five months.

## Discussion

MRT is a rare and highly aggressive malignancy, typically afflicting children under the age of three to four, with an average diagnosis occurring at 15 months of age [6]. In the United States, an estimated 20 to 25 new cases are diagnosed annually [9]. The primary site of MRT varies, with the central nervous system: atypical teratoid rhabdoid tumor (ATRT) being the most prevalent (~50%), followed by the kidney (~16%), and other locations (extracranial extrarenal MRT) as reported by the Surveillance, Epidemiology, and End Results database (SEER) [2]. Extracranial MRT exhibits a more favorable five-year overall and disease-free survival compared to ATRT, attributed to a higher rate of complete surgical resection, radiotherapy, and an older age at diagnosis. Advanced stage and younger age at diagnosis are recognized as risk factors for prognoses in extracranial MRT [6-7]. Among extracranial MRT, the MRT of the kidney demonstrates a lower survival rate (five-year overall survival of 36.5%) compared to extracranial extrarenal MRT (50.1%) [10].

Initial clinical and radiological findings necessitated a differential diagnosis of Kaposiform hemangioendothelioma and epithelioid angiosarcoma. Histologically, both entities express endothelial markers such as CD34. Kaposiform hemangioendothelioma exhibits a mix of vascular nodules and spindle cells, with features overlapping with benign hemangioma and Kaposi sarcoma. Neoplastic cells demonstrate minimal cytologic atypia with a pale eosinophilic cytoplasm [11]. Epithelioid angiosarcoma, a variant of angiosarcoma, displays round or polygonal epithelioid cells with abundant eosinophilic cytoplasm, vesicular nuclei, and prominent nucleoli [12]. *GNA14* mutations have been reported in some Kaposiform hemangioendothelioma cases, while various mutations are found in angiosarcomas.

The congenital disseminated pattern of presentation makes the identification of the primary tumor site or potential familial cases associated with rhabdoid tumor predisposition syndrome (RTPS) difficult. RTPS, a rare and aggressive genetic disorder, is characterized by an increased susceptibility to developing rhabdoid tumors, predominantly affecting younger children. Caused by mutations in the *SMARCB1* (*INI1*) gene, RTPS often predisposes individuals to early-onset rhabdoid tumors within various organs [13]. While the detection of *SMARCB1* mutations in the somatic cells of the patient or his parents could have highlighted the presence of RTPS, such data was not collected.

## Conclusions

Congenital MRT presents with a poor prognosis, often leading to fatal complications. The majority of MRT cases involve mutations in the *SMARCB1* or *SMARCA4* genes, necessary for diagnosis. The overall incidence of MRT is estimated to be around 0.5 per million children, and it tends to manifest early in life, reaching a peak incidence within the first year. Distinguishing MRT from other soft-tissue sarcomas poses considerable challenges, underscoring the importance of histopathologic and immunohistochemistry tests in conjunction with clinical and radiologic findings.

The rarity of MRT presents considerable challenges in both research and clinical management. Collaborative efforts are essential to developing effective therapeutic strategies for this aggressive pediatric cancer. This case of congenital disseminated MRT is presented due to its rarity, dismal prognosis, and the pressing need for timely intervention. Despite chemotherapy improving the arm lesion, the continuous progression of lesions in bones, lungs, liver, adrenal gland, and kidneys suggests a heterogeneous tumor component and variable treatment response. This contribution aims to expand the case pool, fostering future mechanistic studies to enhance our understanding and treatment options for this challenging and often fatal disease.
